# Evaluation of the Visual Stimuli on Personal Thermal Comfort Perception in Real and Virtual Environments Using Machine Learning Approaches

**DOI:** 10.3390/s20061627

**Published:** 2020-03-14

**Authors:** Francesco Salamone, Alice Bellazzi, Lorenzo Belussi, Gianfranco Damato, Ludovico Danza, Federico Dell’Aquila, Matteo Ghellere, Valentino Megale, Italo Meroni, Walter Vitaletti

**Affiliations:** 1 Construction Technologies Institute, National Research Council of Italy (ITC-CNR), Via Lombardia, 49, 20098 San Giuliano Milanese, Italy; bellazzi@itc.cnr.it (A.B.); belussi@itc.cnr.it (L.B.); danza@itc.cnr.it (L.D.); ghellere@itc.cnr.it (M.G.); meroni@itc.cnr.it (I.M.); 2 SCS, Softcare Studios Srls, Via Franco Sacchetti, 52, 00137 Roma, Italy; g.damato@tommigame.com (G.D.); info@softcarestudios.com (V.M.); 3 VIGAMUS Academy, Università degli Studi Link Campus University, Via del Casale di San Pio V, 44, 00165 Roma, Italyw.vitaletti@gmail.com (W.V.)

**Keywords:** indoor thermal comfort, personal thermal comfort perception, wearable, internet of things (IoT), machine learning, virtual reality

## Abstract

Personal Thermal Comfort models consider personal user feedback as a target value. The growing development of integrated “smart” devices following the concept of the Internet of Things and data-processing algorithms based on Machine Learning techniques allows developing promising frameworks to reach the best level of indoor thermal comfort closest to the real needs of users. The article investigates the potential of a new approach aiming at evaluating the effect of visual stimuli on personal thermal comfort perception through a comparison of 25 participants’ feedback exposed to a real scenario in a test cell and the same environment reproduced in Virtual Reality. The users’ biometric data and feedback about their thermal perception along with environmental parameters are collected in a dataset and managed with different Machine Learning techniques. The most suitable algorithm, among those selected, and the influential variables to predict the Personal Thermal Comfort Perception are identified. The Extra Trees classifier emerged as the most useful algorithm in this specific case. In real and virtual scenarios, the most important variables that allow predicting the target value are identified with an average accuracy higher than 0.99.

## 1. Introduction

The design of comfortable indoor environments in buildings is topical as users spent much of their time indoors [1,2]. The scientific community dealt with the Indoor Environmental Quality (IEQ) for decades, with a considerable amount of researches and studies, and this interest continues today [3]. These works have shown how IEQ directly affects the comfort, health, and productivity of occupants [1] besides energy use [4]. The weight of IEQ in building design is stated by the international and national standards. Recently, the European Directive on the energy efficiency put energy issues and occupants’ well-being on equal footing [5].

IEQ is a holistic concept that includes aspects related to architecture, Heating, Ventilation and Air Conditioning (HVAC) design, Thermal Comfort (TC), Indoor Air Quality (IAQ), lighting, acoustics, and control systems [6,7]: studies showed that TC is the most important factor, especially in workplaces [8].

TC has been widely analyzed over the years [6], with the result that standards and models have become commonplace among professionals. Despite almost a century having passed since the first studies on TC [7] and 50 years since the revolutionary works of Povl Ole Fanger [8], the issue related to TC is still topical and engages the scientific community with many initiatives (for example, IEA Annex 79: Occupant-Centric Building Design and Operation [9]). Efforts are aimed at understanding the complex bidirectional interaction between occupants and building technologies and at defining new models that fill the gap of the current methodologies to design comfortable, usable, adaptable, and energy-efficient buildings [10]. New technologies and approaches, such as Internet of Things (IoT), Virtual Reality (VR), and Machine Learning (ML) techniques [11,12,13] are applied to face this issue.

In recent years, the approach to TC has switched from an average response of large population to a Personal Thermal Comfort Perception (PTCP) predicting an individual’s thermal comfort response [12]. The development of such new paradigms requires the analysis of a great amount of data related to the environment, the human condition, perception, etc. The management of these data needs the use of specific devices and tools for their detection and computation. The resources made available by the IoT and ML techniques are exploited for this goal [14]. Thermal Comfort Models ((TCMs) are built based on pervasive collection and analysis of this data and their relationships with the users’ thermal perception of the indoor climate considering a wide spectrum of variables, related both to users and the indoor environment. Compared to the classic treatment of the study of TC, personal TCMs require the use of an advanced algorithm that is able to manage a large amount of data; from this perspective, ML techniques are showing their potential. Several authors have investigated this research domain through field studies involving occupants in different thermal configurations. In [15], a personalized HVAC control framework based on the Random Forest Classifier (RFC) is proposed integrating environmental and human physiological and behavioral data. In [16], the authors state that the most accurate model to predict the personal thermal comfort of occupants is the Classification And Regression Trees (CART). The research conducted in real offices involving eight workers detects human and environmental variables with IoT solutions. In [17], ML techniques are applied to identify the most relevant parameters for users’ recognition. In [18], the Bagging model shows higher accuracy than Support Vector Machine (SVM) and Artificial Neural Network (ANN) in thermal perception prediction. In [19], the effectiveness of a personal thermal sensation modeling method based on the C-Support Vector Classification algorithm has been verified. In [20], the SVM algorithm has been compared with several other popular machine learning algorithms to define thermal comfort perception. In [21], an intelligent control method based on a SVM classifier is proposed. In [22], a personalized classification model was developed using the least-squares SVM algorithm. If on the one hand these researches prove the reliability of ML techniques in the assessment and prediction of users’ TC, on the other hand, it is not possible to provide a rank of the algorithms due to the variability of data and situations.

Today, VR is reaching almost all sectors; the building sector is no stranger to this evolution. Researchers investigated the potential of this technology in specific fields such as energy efficiency [23] and TC [24], finding interesting perspectives for future developments. VR is a promising approach for investigating the interactions between users and indoor environments, allowing an analysis of the aspects related to IEQ. In this domain, research studies have been carried out demonstrating how VR is suitable to simulate a real environment’s features and to investigate the user’s perception in relation with the indoor environment [25], even if any differences in the human body response can occur between real and virtual environments [26]. As a cognitive technology specifically targeting human perception, it also represents a useful tool to highlight the relative contribution of visual perception in PTCP, allowing to separate this aspect from the complexity of the surrounding environment. There are studies in the literature about the assessment of thermal perception in VR. Some of them are aimed at evaluating the differences on users’ perception between real and virtual environments and the possible causes of this difference [27]. Other research studies assess the effect of the exposure to a particular situation on human perception in VR, such as for example the effect of colors on people’s perception of TC [28,29].

Taking into account the complexity of this domain, the present article aims at investigating the potentialities of the combination of IoT and ML in analyzing and predicting the TC of users in different scenarios. In particular, real (R) and virtual (VR) scenarios were set up, and 25 users were involved in the experimental campaign, so that any differences in the perception of TC can be identified. For this purpose, two settings with different light colors (red and blue) chosen considering the experience of Fanger [30] were considered. This area of research was originally studied by Bennet [31] and Fanger [30], finding that this effect on human comfort has no practical significance. Most recently, studies have questioned these outcomes, detecting a relationship between color and thermal sensation with a positive effect on energy consumption [32,33,34,35,36]. The exploitation of advance technologies of visualization and modeling or the real environment such as VR can contribute to a shared outcome. In the article, light color is an input variable of the dataset used along with environmental and personal variables by the considered ML models to define the PTCP.

The main contribution of the paper regards the possibility of comparing some ML algorithms in in two different scenarios, defining the most important variables to define PTCP.

The second chapter describes the workflow followed to define the dataset with a description of the used devices. The third chapter reports the outcomes obtained with the ML techniques.

## 2. Materials and Methods

### 2.1. Methodological Approach

**Figure 1:**
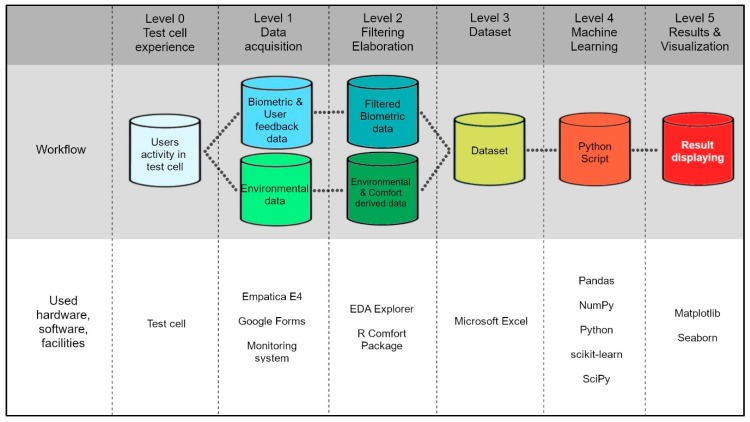
Overview of the workflow and hardware, software, and facilities used to create the dataset.

The experimental campaign is carried out in a test cell where 25 participants are asked to express their thermal satisfaction under R and VR scenarios. The detected data were managed with ML techniques for PTCP recognition according to the methodological framework described in Figure 1.

Participants alternated to R and VR scenarios within the test cell during eight non-consecutive days (Level 0). Each user performed the experience in both scenarios at the same time of the day (morning or afternoon) in order to minimize the influencing effects due to the circadian rhythm. Data are detected through a pervasive monitoring system consisting of environmental and wearable sensors (Level 1): biometric data are recorded through the Empatica E4 wristband [37], users’ feedbacks are recorded through a web multiplatform survey based on a Google Form model, and the environmental data are detected through a monitoring system as described in detail in Section 2. The biometric data are processed using a noise detection ML algorithm that allows automatically detecting Electrodermal Activity (EDA) artefacts [38,39] (Level 2). For example, the noise in a wearable device may be due to the excessive movement or adjustment of the device. Raw data recorded with a sampling frequency of 4 Hz are divided into periods of 5 s and then filtered considering a noise classification number (Binary Labels) equal to −1 (noise data) or 1 (clean data). Besides, all data are used for a parametric analysis through a specific comfort package, “comf”, which was developed for the R environment [40,41,42] to calculate the following thermal indices:

- Predicted Mean Vote (PMV).
- PMV_MetBMR_, the Predicted Mean Vote (PMV) adjusted considering the Basal Metabolic Rate (BMR) of each participants.
- dTNZ, the distance to ThermoNeutral Zone (dTNZ) [40] defined in a Cartesian orthogonal reference system with ambient temperature in X-axis and skin temperature (Tskin) in the Y-axis, as the distance from the band defined as the “range of ambient temperature at which thermal regulation is achieved only by control of sensible (dry) heat loss” [15].

The environmental data and user feedbacks were merged with the filtered biometric dataset defining the complete dataset (Level 3) used for the analysis with the ML approach (Level 4). Then, the results of the analysis are displayed (Level 5).

### 2.2. Test Cell, Monitoring System, and Participants

**Figure 2:**
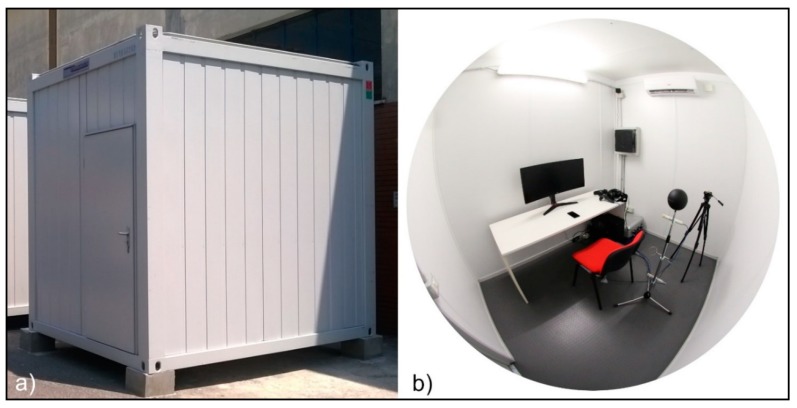
Test cell: (**a**) outdoor photo; (**b**) fisheye indoor photo.

The test cell, located within the headquarters of ITC-CNR near Milan, is an industrial container (300 cm length × 250 cm width × 300 cm height) without windows (Figure 2) that is East–West oriented and properly insulated in accordance with the Italian regulation in force [43].

**Figure 3:**
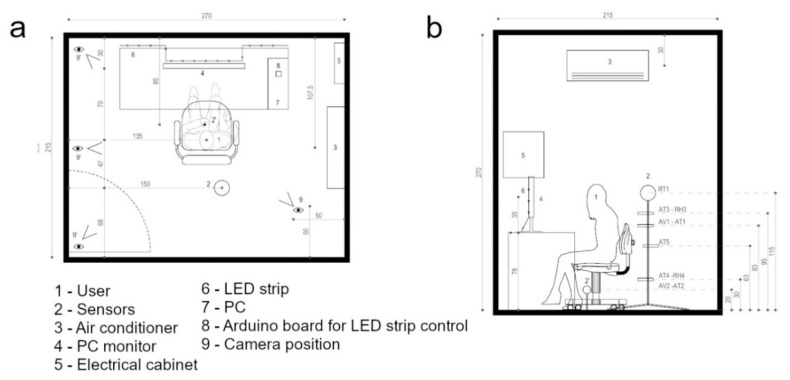
Distribution of the hardware in the test cell: (**a**) plant; (**b**) cross-section.

A single workstation is installed within the cell with the following devices:

- a smartphone, only in R scenario to record the users’ feedback;
- the monitoring system;
- a VR headset, only in the VR scenario;
- an RGB strip LED installed on the rear of the monitor and in the upper edge of the desktop, as defined by a preliminary study with Radiance;
- an Arduino board connected to a TSOP31238 IR receiver (Vishay, Selb, Germany) and an LED IR-type (8 in Figure 3). This system records, through the reverse engineering process, the codes that the remote control sends to the 150 SMD5050 RGB (Tomshine, Guangzhou, China) strip LED [44]. An IR LED emitter manages the lighting (6 in Figure 3).

A thermorugulator Vemer HT NiPT-1 (Vemer, Villapaiera di Feltre, Italy) guarantees a temperature set-point of 21 °C (±0.5). It is connected to a platinum resistance Pt100 positioned in the middle of the test cell (AT5 in Figure 3) that is able to provide the air temperature.

To ensure the required comfort level, according to the category “B” defined in EN ISO 7730, through the Computational Fluid Dynamics (CFD) simulations ([45,46]), the optimal flow rate (330 m^3^ h^−1^), angle of rotation of the deflector, and position of the user were defined.

The monitoring system (Figure 3) consists of two thermo-hygrometric sensors, a black globe thermometer, and two hot wire anemometers. All sensors are connected to a 32-bit ARM data logger that records the monitored values with a 5-s detection frequency. All participants wear two smart wristbands Empatica E4, which are installed on both the arms. It integrates a Photoplethysmography (PPG) sensor for the detection of the Heart Rate (HR), an EDA sensor, an infrared thermopile for the measurement of Tskin, and a three-axis accelerometer. 

Table 1 reports the characteristics of the sensors for the monitoring of the indoor environmental variables. The AT5 thermometer is used for the actuation system and not for monitoring purposes.

The Google Forms web-based survey is defined according to the guidelines provided by the Standard ANSI/ASHRAE 55:2017 [44]. Table 2 reports the survey questions that participants are required to fill out. As reported in the table, the question about thermal perception is based on the seven-point ASHRAE scale, while questions about the satisfaction to different environmental conditions are based on a Likert Scale from 1 (very satisfied) to 7 (very dissatisfied).

The test is divided into three parts. After a period of acclimatization of 20 min [47], participants are invited to answer a first questionnaire related to their personal characteristics (Q1) and their thermal sensation (Q2). Users are invited to answer Q2 even in the mid-point and at the end of the experience, before leaving the test cell. The analysis of Q1 allowed the calculation of the thermal resistance of the clothing in compliance with Annex C of the Standard EN ISO 7730 [48]. An additional thermal resistance of 0.1 clo for sedentary activities due to the standard office chair is considered [49], while extra thermal insulation due to the visor and headphones is not considered. In Table 3, the considered average clothing insulation value is reported. Other information available through the web-based survey allow to identify the metabolic rate [50,51,52,53] and the thermal sensation of the individuals. 

In compliance with the mean value of subjects involved in experiments related to the analysis of TC found in literature [54], a sample of 25 participants was chosen: the aggregated data of users are listed in Table 3. All users are from South Europe: 12 females and 13 males. They represent a heterogeneous pattern of testers considering the following variables: sex, age, weight, and height. All subjects previously provided their informed consent for inclusion.

The standard metabolic rate Met_st_ is defined in accordance with the value reported in Annex B of EN ISO 7730 [48].

The Met_BMR_ parameter in met is defined starting from the BMR for each participant, which was calculated according to the equation defined by Mifflin [55] as a function of weight, height, age, and gender.

### 2.3. Experimental Design

**Figure 4:**
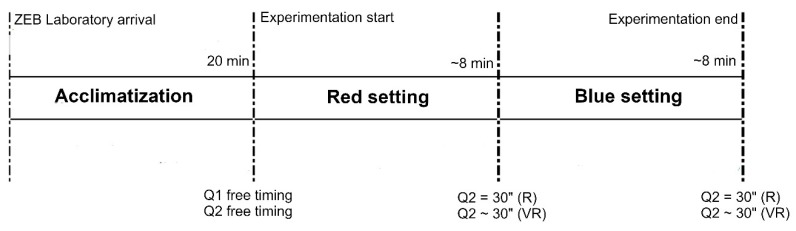
Timing of the experimentation.

During the experience, the participants are immersed first in an R scenario and then, after an interval of about 45 min, in a VR scenario. Each experiment lasted on average 17 min (Figure 4), excluding the initial acclimatization period. As better described below, all users are exposed to two type of diffused colored light: red and blue, according to Fanger [30].

**Figure 5:**
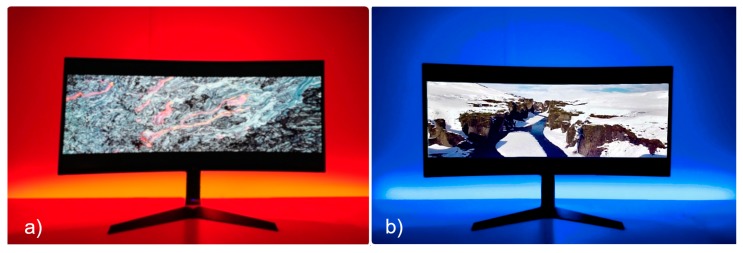
Real scenario: (**a**) red setting; (**b**) blue setting.

In the R scenario, the participant watches a 16-minute video that contains in the first 8 min scenes such as volcanoes evoking heat sensations while the LED strip is red (Figure 5a). At the halfway point of the video, the LED turns into neutral light and the participant fills out questionnaire Q2. Then, the participant watches the second half of the video containing scenes of cold sensations (snow, glaciers) while the LED strip is blue (Part III, Figure 5b). At the end of the video, the LED returns to neutral and the user fills out questionnaire Q2 again.

**Figure 6:**
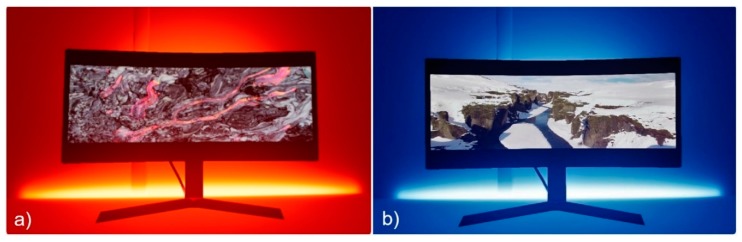
Virtual scenario: (**a**) red setting; (**b**) blue setting.

The VR is experienced through a HTC Vive headset (HTC, Taoyuan, Taiwan) [56] and designed using the graphical Unreal Engine 4 [57] for the real reproduction of all parts of the scene, video playing, and light color changes (Figure 6). A virtual questionnaire was designed to collect users’ feedback without interrupting the immersion in the VR scenario while a wireless motion controller permits filling in the questionnaire. A few dynamic point lights are created, which were placed at the main lighting sources with no use of the baked light. The deferred rendering method is used for this purpose. The Illuminating Engineering Society (IES) light profiles are used to increase the reliability of the lights with bulbs used in the room, and the transition of the RGB LEDs in the various phases of the experience are synchronized to the video through the unreal editing tools. A high level of detail was dedicated to near objects, and a video frame rate of 90 fps was used to influence the participants’ perception. Finally, to prevent performance drops, few material textures are used and small elements such as LED strips and distant details are avoided since they are not in any case visible to the end user.

### 2.4. Dataset Attributes

A preliminary dataset composed by 22,575 instances (rows) and 30 attributes structured as reported in Table 4 have been collected. All biometric and environmental data with *Binary Labels* values equal to 1 (filtered data) are considered. The PMVs and dTNZ values are defined considering the comfort package for the R programming language environment.

The ML approach allows defining the most influential factors, among the biometric (features from 0 to 5) and environmental data (features from 13 to 26), that affect the PTCP. Feature 6 is used as a filter. Features 7 to 10 are categorical labels used to identify the sub dataset. In particular, Feature 10, SXvsDX, is applied to verify differences in terms of biometric data acquired with the two wearables on the dominant and non-dominant hand: the manufacturer recommends wearing the smart band on the non-dominant wristband [58], while recent studies show that the dominant side may have a much stronger EDA signal [59]. The pre-assessment displays a non-remarkable difference between the biometric data acquired on the two wrists. This condition is also verified by [60]. The asymmetries between skin conductance measurements on the left and the right side is not the key point of this research, but considering the so-structured dataset, it could be considered in future studies. Features 11 and 12 are the target values. Features 27, 28, and 29 are used to define a correlation factor with PTCP.

## 3. Results

### 3.1. Dataset Preliminary Analysis 

**Figure 7:**
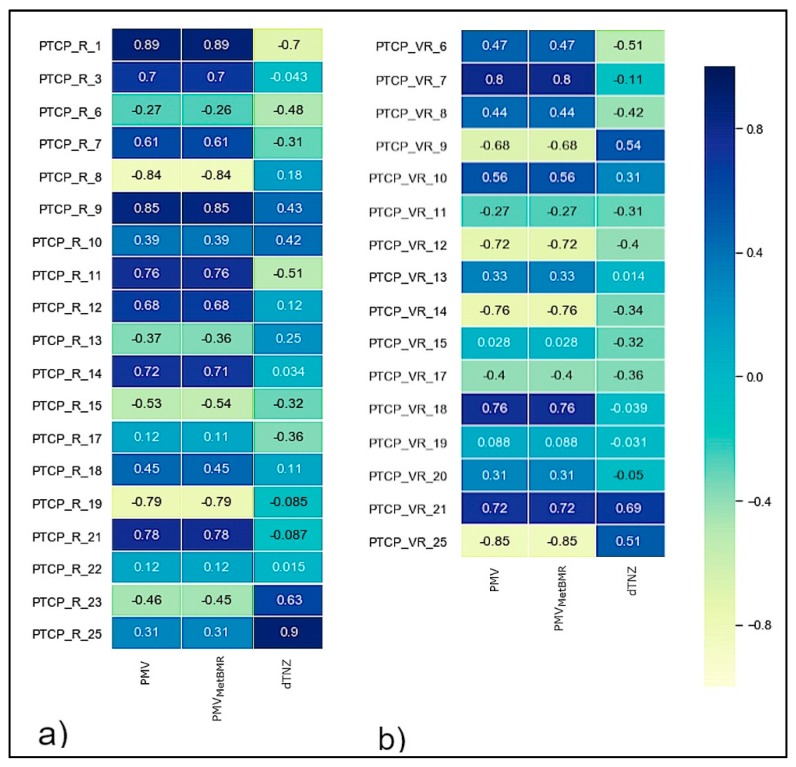
Confusion correlation matrix of: (**a**) PTCP_R; (**b**) PTCP_VR.

The whole dataset has been subdivided in two sub-datasets as a function of the categorical label RvsVR according to the real or virtual environment. The correlation between the considered standard comfort models (PMV, PMV_MetBMR_, dTNZ) and the PTCP of the participants is investigated. Figure 7 shows the Pearson correlation index for both PTCP_R and PTCP_VR, showing the direct or indirect relationship of the users’ PTCP with respect to the considered thermal models. 

Some users’ data are not present in the scenarios. This fact is due to:

- for some users, there are no “cleaned” data;
- in case of zero variance of PTCP, no correlations could be defined (division by zero).

The correlation between the standard thermal models with the PTCP of participants can be considered “moderate” (0.3–0.7) or “strong” (>0.7) following the Pearson classification [61]. Besides, the dTNZ model presents a “weak–moderate” correlation (0.0–0.7). The linear correlation is positive or negative depending on the considered user, and it is therefore impossible to define an average behavior for the considered models. In terms of absolute values, the PMV and PMV_MetBMR_ models have on average higher values of correlation if compared with the dTNZ model both in real and virtual environments.

### 3.2. Machine Learning Techniques Application and Final Results

A set of six algorithms are considered for the determination of the target value (PTCP_R or PTCP_VR): Linear Discriminant Analysis (LDA) [62], Logistic Regression (LR) [63], Decision Tree Classifier (CART) [64], Extra Tree Classifier (ETC) [65], Linear Support Vector Classifier (LSVC) [66], Random Forecast Classifier (RFC) [67]. For each algorithm, the default parameters reported in the references are considered for the analysis.

The whole dataset is split into two subsets: the former, composed by 60% of the available data, is used for the training, while the latter, with the remaining 40%, is used for the validation. Depending on the number and the type of considered features reported in Table 5 and Table 8, all instances are considered as an input variable “x”, and the instances of attribute 11 or 12 (PTCP_R or PTCP_VR) are considered as the target variable “y”. The metric of *Accuracy* [68] is used to evaluate the models, and it is defined as the ratio of the number of correctly predicted instances divided by the total number of instances in the dataset. The k-fold [69] cross-validation (n_splits = 10, shuffle = False, random_state = 7) is used to evaluate the performance of the different algorithms. 

Considering the first subset where PTCP_R attribute is not a “NaN”, the average (*Avg*) accuracy and the standard deviation (±std) for each algorithm is defined (Table 5).

Recursive Feature Elimination (RFE) [70] is used to detect the importance of the individual features in the definition of the target value PTCP_R considering the number of the most important features that, starting from 22, is halved at every subsequent step (Table 5).

As reported in [71], tree-based models always work better than the alternatives when there is no hyperparameter tuning. To verify this circumstance, the tuning of the hyperparameters was carried out for LR and LSVC, and Table 6 shows the hyper parameters tuned and their corresponding ranges. LDA has not been considered because it has no hyperparameter to tune [62].

ETC with six input features (*Color*, *User*, *RH3*, *AT4*, *T_avg_2* and *RT1*) provides the highest *Avg* accuracy and the lowest ± std. 

The statistical significance of the results is verified using the T-test SciPy function [71], due to the data of each sample that are normally distributed. Fifteen tests were conducted for each pair of sample combinations. The p-values are lower than 0.05, demonstrating the statistical significance of the results.

**Figure 8:**
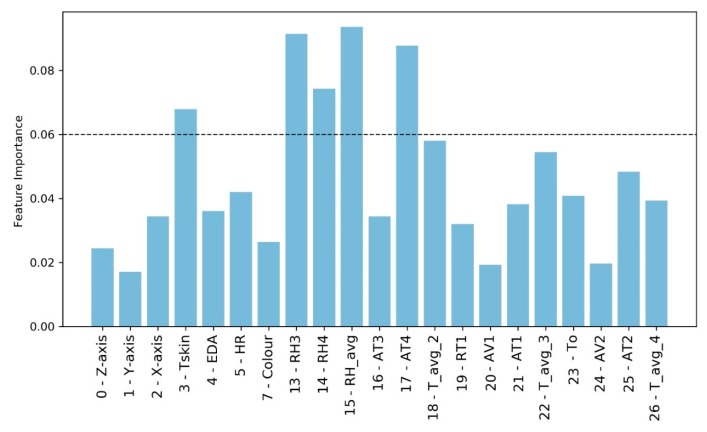
Relative feature importance of the Extra Trees classifier using all the environmental and biometric data to define Users in the R scenario.

The ETC algorithm is used for the following analyses. It is possible to replace the categorical label *Users* by using an Extremely Randomized Tree technique [72] with Python’s *scikit-learn* tool [73]. In this way, it is possible to verify the importance of individual features to identify the target feature, *Users* (Figure 8).

The results obtained considering a threshold feature importance value equal to 0.06 reveal that the data related to the 3D accelerations are not relevant. Besides, EDA and HR are not very significant, while *Tskin* is the only biometric variable that has a considerable weight in *User* definition. Similarly, some environmental variables, in this specific case, are of limited importance in the definition of the users, such as AV, due to low fluctuation. So, it is possible to identify some variables (*Tskin*, *RH4*, *AT4*) that replace the variable *User* in the definition of PTCP_R. In this way, the previous subdataset composed by (*Color*, *User*, *RH3*, *AT4*, *T_avg_2* and *RT1*), which was used to define PTCP_R with the highest *Avg* accuracy considering the ETC model, is replaced by the new consisting of *3-Tskin*, *7-Color*, *13-RH3*, *14-RH4*, *17-AT4*, *18-T_avg_2*, and *19-RT1*.

**Figure 9:**
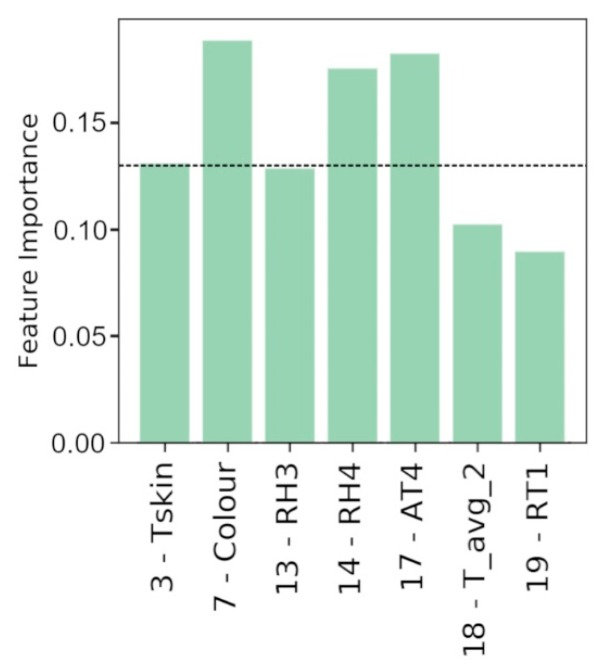
Relative feature importance of Extra Trees classifier using a restricted number of data to define PTCP_R.

Figure 9 reports the relative feature importance in order to identify PTCP_R considering the identified key features. 

Considering the first four variables, with a feature importance greater than 0.13 (*3-Tskin*, *7-Color*, *14-RH4*, and *17-AT4*) the average accuracy (0.997) and related standard deviation (0.003) have been calculated.

Table 7 shows the classification report summarizing the results as a final accuracy score of ETC model directly on the validation set. It shows excellent results in terms of the prediction of PCTP considering four indicators [74].

- Precision defined as a measure of a classifier’s exactness;
- Recall considered as the completeness of the classifier;
- F1-score, a weighted average of precision and recall;
- Support, the number of occurrences of each label in y are true.

The same approach has been carried out considering a second sub-dataset obtained considering the data of the VR scenario only. In this case, the RFE allows identifying the importance of features to define PTCP_VR (Table 8).

**Figure 10:**
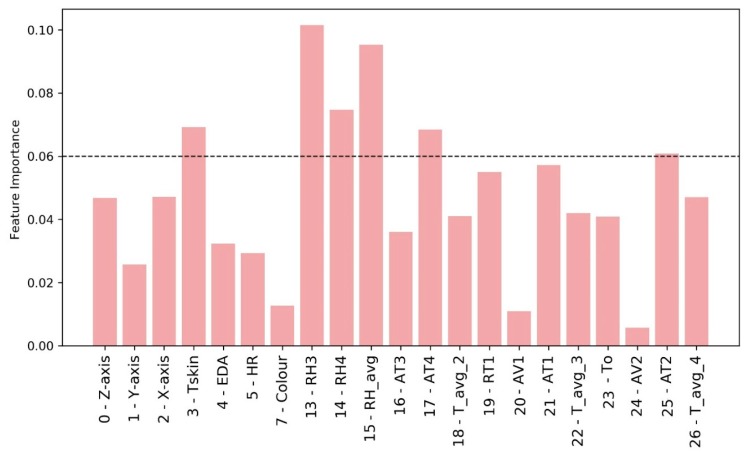
Relative feature importance of Extra Trees classifier using all the environmental and biometric data to define users in the VR scenario.

The statistical significance of the results is verified also considering the above reported VR results. ETC with six six input features (*Color*, *User*, *RH3*, *RH4*, *T_avg_2*, and *AT2*) provides the highest *Avg* accuracy (0.997) and the lowest ±std 0.002. It is possible to verify the importance of individual features in order to identify the categorical label Users (Figure 10) using the same approach as that for the R scenario.

**Figure 11:**
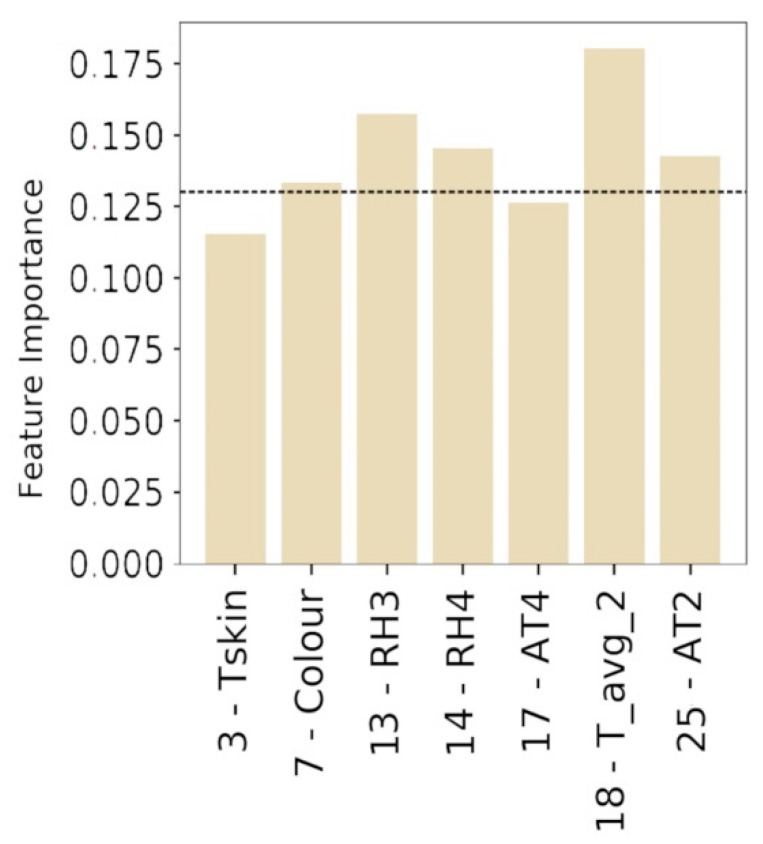
Relative feature importance of Extra Trees classifier using a restricted number of data in order to define PTCP_VR.

In addition, in the virtual scenario, Tskin is the only individual variable that has a significant weight in User definition. *Tskin*, *RH3*, are *AT4* are suitable to replace the variable *User* in the definition of PTCP_VR. The new dataset used to define PTCP_VR consists of *3-Tskin*, *7-Color*, *13-RH3*, *14-RH4*, *17-AT4*, *18-T_avg_2*, and *25-AT2*. Figure 11 reports the relative feature importance to predict the PTCP_VR considering the identified key features. 

Considering the first five variables, with a feature importance greater than 0.13 (*7-Color*, *13-RH3*, *14-RH4*, *18-T_avg_2*, *25_AT2*), the average accuracy (0.995) and its standard deviation (0.004) have been calculated.

Table 9 shows the classification report summarizing the results as a final accuracy score of the ETC model directly on the validation set. It shows excellent results in terms of the prediction of PCTP considering the four indicators introduced previously [74].

## 4. Conclusions

Even if several studies successfully confirmed the effectiveness of using the ML approach to define PTCP, they fail to analyze the influence of the selected variables in a virtual scenario: it was unclear whether there was a difference in PTCP modeling in real and virtual environments. Based on this domain of investigation, the proposed approach describes research where TC is evaluated considering ML algorithms, smart devices, and VR. In particular, VR was applied to analyze which variables could affect the thermal perception of individuals immersed in a virtual environment as realistically as possible. For this purpose, light colors were used as an endogenous variable, and two configurations with red and blue colors were designed.

The outcomes of this research show how, in R and VR scenarios with comparable indoor environmental variables, the light color is a non-negligible factor in predicting thermal perception.

Under the experimental conditions, the classical TCMs (PMV, PMV_MetBMR_, and dTNZ) show a variable correlation with respect to the PTCP. The use of ML techniques allows improving this correspondence. Among the considered ML algorithms, ETC is the most suitable model in terms of the average accuracy score. A second important result is that with the application of the ML approach in both R and VR scenarios, T-Skin is the physiological variable that much more affects the accuracy of the model in terms of PTCP prediction. Other personal data such as EDA and HR do not have a considerable impact on the thermal sensation. This could be related to the sedentary activity performed by participants during the test that does not produce significant variation in these variables.

Third, the opportunity to assess PTCP using standardized VR scenarios facilitates the collection of a wider amount of sharable and comparable data, allowing a deepening of the study of TC in different indoor settings. The outcomes of such analysis result in a variety of real-life applications. It also allows isolating the subjective perceptual cues impacting users’ PTCP in a percentage still to be characterized and quantified. Once available, the aforementioned aspects could provide actionable insights for innovative strategies and interventions aiming at PTCP improvement.

A data-based approach to indoor comfort is a fundamental prerequisite for designing a library of the best settings and conditions to boost personal comfort intended as a necessary condition to support personal, social, and working performances. VR scenarios enable the opportunity to speed up the whole process, reducing the required resources to build traditional analog settings. Introducing VR environments also increases the complexity of collectible data, allowing for a deeper analysis and correlation between PTCP, the provided sensorial content, and users’ behavior.

However, to maximize the potentials of this approach, some limitations emerged during the experimentation must be overcome.

Firstly, the experimentation has been carried out in a laboratory test cell, which is not representative of a real working environment: in this context, it is difficult to scale the results to a real building. Comparing the obtained results in R and VR, it is possible to upgrade the results of the experimentation developing new virtual scenarios that differ from the real base case.

A second potential limit is that we did not consider the color’s psychological influence on PTCP [75].

Finally, the approach can be improved considering a wider set of ML models, even if the chosen model has produced satisfactory results.

## Figures and Tables

**Table 1:** Characteristics of sensors installed in the test cell. EDA: ElectroDermal Activity; HR: Heart Rate; PPG: Photoplethysmography; Tskin: skin temperature; RH: Relative Humidity; AT: Air Temperature; RT: Radiant Temperature; AV: Air Velocity.

Sensor	Variable	ID in Figure 3	Measure Range	Accuracy
Thermo-hygrometer	RH	RH3, RH4	0–100%	±2%
Thermo-hygrometer	AT	AT3, AT4	−40 to +60 °C	±0.1 °C
Black globe thermometer	RT (derived)	RT1	−40 to +60 °C	±0.1 °C
Hot wire anemometer	AV	AV1, AV2	0–5 m/s	±0.02 m/s
Hot wire anemometer	AT	AT1, AT2	−20 to +80 °C	±0.3 °C
Thermometer	AT	AT5	−70 to +500 °C	-
PPG sensor	HR (derived)	-	-	-
EDA sensor	EDA	-	0.01–100 µS	-
Skin temperature sensor	Tskin	-	−40 to +85 °C	-
3-axes accelerometer	Accelerations	-	±2 g	-

**Table 2:** Questions of the web-based survey.

Questionnaire	Experience Period	Questions	Answer Options
Q1	After the acclimatization, at the beginning of the test (Part I)	User	1 to 25
		Height	Value in [cm]
		Weight	Value in [kg]
		Age	Value in [y]
		Gender	Female, Male
		Clothing worn right now?	T-shirt, Long-sleeved shirt, Shirt, Long-sleeved sweatshirt, Sweater, Jacket, Light skirt, Heavy skirt, Light-weight trousers, Normal trousers, Flannel trousers, Slip, Ankle socks, Long socks, Nylon stockings, Thin-soled shoes, Thick-soled shoes, Boots, Other
Q2	At the beginning (Part I), in the middle (Part II), and at the end of the test (Part III)	Thermal sensation perceived?	Cold, Cool, Slightly cool, Neutral, Slightly warm, Warm, Hot
		How satisfied are you with the humidity of the indoor environment?	(Very satisfied) 1 to 7 (Very dissatisfied)
		How satisfied are you with the indoor air speed?	(Very satisfied) 1 to 7 (Very dissatisfied)
		How satisfied are you with the indoor temperature?	(Very satisfied) 1 to 7 (Very dissatisfied)
		Describe your current emotional state	Relaxed, Happy, Sad, Angry, Agitated

**Table 3:** Aggregated data of the 25 participants involved in the test.

Age [y]	Weight [kg]	Height [cm]	Iclo [clo]	Met_st_ [met]	Met_BMR_ [met]
Avg ± std	Avg ± std	Avg ± std	Avg ± std	Avg ± std	Avg ± std
45.12 ± 9.36	69.30 ± 16.09	171.64 ± 7.43	0.94 ± 0.09	1.20 ± 0.00	1.42 ± 0.13

**Table 4:** Dataset attributes. VR: Virtual Reality.

Number	Data Label	Description	Unit	Number of Non-Null Value	Number of Data for which BL = 1
0	Z-axis acceleration	acceleration along the Z-axis	[g]	22,575	14421
1	Y-axis acceleration	acceleration along the Y-axis	[g]	22,575	14,421
2	X-axis acceleration	acceleration along the X-axis	[g]	22,575	14,421
3	Tskin	Skin temperature	[°C]	22,575	14,421
4	EDA	Electrodermal activity	[μS]	22,575	14,421
5	HR	Heart rate	[bpm]	22,575	14,421
6	Binary Labels	Classification label	-	22,575	14,421
7	Color	Setting of the environment	-	22,575	14421
8	User	Number identifying the user	-	22,575	14,421
9	RvsVR	Type of setting: Real or VR	-	22,575	14,421
10	SXvsDX	Biometric origin: left or right smartband data	-	22,575	14,421
11	PTCP_R	Personal Thermal Comfort Perception in real environment	-	11,347	7295
12	PTCP_VR	Personal Thermal Comfort Perception in virtual reality	-	11,228	7126
13	RH3	See Table 1	[%]	22,575	14,421
14	RH4	See Table 1	[%]	22,575	14,421
15	RH_avg	Average value between RH3 and RH4	[%]	22,575	14,421
16	AT3	See Table 1	[°C]	22,575	14,421
17	AT4	See Table 1	[°C]	22,575	14,421
18	T_avg_2	Average value between AT3 and AT4	[°C]	22,575	14,421
19	RT1	See Table 1	[°C]	22,575	14,421
20	AV1	See Table 1	[m/s]	22,575	14,421
21	AT1	See Table 1	[°C]	22,575	14,421
22	T_avg_3	Average value among AT3, AT4, and AT1	[°C]	22,575	14,421
23	To	Operative temperature defined as the average value between RT1 and T_avg_4	[°C]	22,575	14,421
24	AV2	See Table 1	[m/s]	22,575	14,421
25	AT2	See Table 1	[°C]	22,575	14,421
26	T_avg_4	Average value among AT1, AT2, AT3, and AT4	[°C]	22,575	14,421
27	PMV	Predicted Mean Vote	-	22,575	14,421
28	PMV_MetBMR_	PMV defined considering specific Met values	-	22,575	14,421
29	dTNZ	Distance to ThermoNeutral Zone	-	22,575	14,421

**Table 5:** Recursive Feature Elimination for R scenario. The feature numbers are the same as those in Table 4, which are reported here for your convenience: **0**: Z-axis acceleration; **1**: Y-axis acceleration; **2**: X-axis acceleration; **3**: Tskin, **4**: EDA; **5**: HR; **7**: Color; **8**: User; **13**: RH3; **14**: RH4; **15**: RH_avg; **16**: AT3; **17**: AT4; **18**: T_avg_2; **19**: RT1; **20**: AV1; **21**: AT1; **22**: T_avg_3; **23**: To; **24**: AV2; **25**: AT2; **26**: T_avg_4. With *: average accuracy defined considering the tuning of hyperparameters. LDA: Linear Discriminant Analysis, LR: Logistic Regression, CART: Decision Tree Classifier, ETC: Extra Tree Classifier, LSVC: Linear Support Vector Classifier, RFC: Random Forecast Classifier.

	22 Features	Accuracy	11 Features	Accuracy	6 Features	Accuracy	3 Features	Accuracy
**Algorithms**		**Avg** ± std		**Avg** ± std		**Avg** ± std		**Avg** ± std
LDA	**[0, 1, 2, 3, 4, 5, 7, 8, 13, 14, 15, 16, 17, 18, 19, 20, 21, 22, 23, 24, 25, 26]**	**0.712** ± 0.018	**[1, 7, 16, 17, 18, 19, 20, 21, 22, 23, 24]**	0.564 ± 0.023	**[16, 19, 20, 22, 23, 24]**	0.507 ± 0.026	**[20, 23, 24]**	0.499 ± 0.036
LR		0.716* ± 0.016	**[0, 1, 2, 7, 16, 17, 18, 19, 20, 21, 23, 25]**	0.581 ± 0.024	**[16, 17, 18, 19, 20, 21, 23]**	0.511 ± 0.034	**[19, 20, 23]**	0.512 ± 0.034
CART		0.987 ± 0.007	**[3, 4, 5, 7, 8, 14, 17, 18, 19, 20, 21]**	0.993 ± 0.005	**[3, 5, 7, 8, 14, 17]**	**0.996** ± 0.003	**[3, 14, 17]**	0.973 ± 0.005
ETC		0.991 ± 0.004	**[3, 7, 8, 14, 15, 17, 18, 21, 23, 25, 26]**	0.997 ± 0.002	**[7, 8, 13, 17, 18, 19]**	**0.998** ± 0.002	**[8, 14, 25]**	0.977 ± 0.004
LSVC		0.723* ± 0.016	**[0, 1, 7, 16, 17, 19, 20, 21, 23, 24, 25]**	0.449 ± 0.106	**[16, 17, 19, 20, 23, 24]**	0.480 ± 0.057	**[20, 23, 24]**	0.480 ± 0.082
RFC		0.996 ± 0.003	**[3, 7, 8, 13, 14, 15, 17, 18, 21, 25, 26]**	0.998 ± 0.003	**[7, 8, 14, 17, 25, 26]**	**0.998** ± 0.003	**[14, 17, 25]**	0.979 ± 0.008

**Table 6:** Hyperparameters tuning range.

Algorithms	Hyperparameters	Range
LR	Solver Penalty C_value	[‘newton-cg’, ‘lbfgs’, ‘liblinear’] [‘l1’, ‘l2’, ‘elasticnet’, ‘none’] [100, 10, 1.0, 0.1, 0.01]
LSVC	Penalty C_value	[‘l1’, ‘l2’] [100, 10, 1.0, 0.1, 0.01]

**Table 7:** Recursive Feature Elimination for R scenario.

Algorithm	PTCP	Precision	Recall	f1-Score	Support
ETC	–1	0.99	1.00	1.00	763
	0	1.00	1.00	1.00	1424
	1	1.00	1.00	1.00	450
	2	1.00	0.99	0.99	281

**Table 8:** Recursive Feature Elimination for the VR scenario. The feature numbers are the same as those in Table 4, which are reported here for your convenience: **0**: Z-axis acceleration; **1**: Y-axis acceleration; **2**: X-axis acceleration; **3**: Tskin, **4**: EDA; **5**: HR; **7**: Color; **8**: User; **13**: RH3; **14**: RH4; **15**: RH_avg; **16**: AT3; **17**: AT4; **18**: T_avg_2; **19**: RT1; **20**: AV1; **21**: AT1; **22**: T_avg_3; **23**: To; **24**: AV2; **25**: AT2; **26**: T_avg_4. With *: average accuracy defined considering the tuning of hyperparameters.

	22 Features	Accuracy	11 Features	Accuracy	6 Features	Accuracy	3 Features	Accuracy
Algorithms		Avg ± std		Avg ± std		Avg ± std		Avg ± std
LDA	**[0, 1, 2, 3, 4, 5, 7, 8, 13, 14, 15, 16, 17, 18, 19, 20, 21, 22, 23, 24, 25, 26]**	**0.715** ± 0.023	**[0, 4, 16, 17, 19, 20, 21, 22, 23, 24, 26]**	0.605 ± 0.026	**[4, 16, 19, 20, 23, 24]**	0.559 ± 0.028	**[16, 19, 24]**	0.539 ± 0.029
LR		0.756* ± 0.022	**[1, 7, 14, 15, 16, 19, 20, 21, 23, 24, 25]**	0.625 ± 0.015	**[1, 16, 19, 20, 21, 23]**	0.525 ± 0.024	**[16, 19, 21]**	0.517 ± 0.022
CART		0.992 ± 0.004	**[0, 2, 3, 4, 7, 8, 14, 15, 17, 18, 23]**	0.993 ± 0.003	**[2, 4, 7, 8, 15, 17]**	**0.994** ± 0.003	**[8, 15, 17]**	0.981 ± 0.007
ETC		0.994 ± 0.003	**[3, 7, 8, 13, 14, 15, 18, 19, 21, 22, 25]**	0.997 ± 0.003	**[7, 8, 13, 14, 18, 25]**	**0.997** ± 0.002	**[8, 15, 25]**	0.982 ± 0.006
LSVC		0.673* ± 0.019	**[1, 7, 14, 16, 19, 20, 21, 22, 23, 24, 25]**	0.476 ± 0.059	**[16, 19, 20, 21, 23, 24]**	0.439 ± 0.130	**[20, 21, 24]**	0.451 ± 0.065
RFC		0.995 ± 0.004	**[3, 7, 8, 13, 14, 15, 17, 18, 21, 25, 26]**	0.996 ± 0.003	**[7, 8, 13, 15, 17, 21]**	**0.996** ± 0.003	**[8, 15, 17]**	0.984 ± 0.007

**Table 9:** Recursive Feature Elimination for the R scenario.

Algorithm	PTCP	Precision	Recall	f1-Score	Support
ETC	−1	1.00	1.00	1.00	540
	0	1.00	1.00	1.00	1165
	1	1.00	1.00	1.00	1078
	2	0.97	1.00	0.99	68

## Nomenclature

AT	Air temperature, (°C)
AV	Air Velocity, (m/s)
BMR	Basal Metabolic Rate, (ml/kg min)
BMR*	Basal Metabolic Rate, (Kcal/day)
CART	Classification and the Regression Trees
CFD	Computational Fluid Dynamics
dTNZ	Distance to ThermoNeutral Zone
EDA	Electrodermal Activity
ETC	Extra Tree Classifier
HR	Heart Rate
Iclo	Thermal Insulation of Clothing, (m2K/W)
IEQ	Indoor Environmental Quality
IoT	Internet of Things
LDA	Linear Discriminant Analysis
LR	Logistic Regression
LSVC	Linear Support Vector Classifier
Met_BMR_	Metabolic rate defined as a function of BMR (met)
Met_st_	Standard tabular value of Metabolic rate (met)
ML	Machine Learning
PMV	Predicted Mean Vote
PMV_MetBMR_	PMV defined considering specific Met values
PPD	Predicted Percentage of Dissatisfied, (%)
PPG	Photoplethysmography
PTCP	Personal Thermal Comfort Perception
R	Real scenario
RFC	Random Forecast Classifier
RFE	Recursive Feature Elimination
RH	Relative Humidity, (%)
RT	Mean radiant temperature, (°C)
TC	Thermal Comfort
TCM	Thermal Comfort Model
TNZ	ThermoNeutral Zone
Tskin	Skin Temperature, (°C)
VR	Virtual Reality

## References

[1] Al Horr Y., Arif M., Kaushik A., Mazroei A., Katafygiotou M., Elsarrag E. (2016). Occupant productivity and office indoor environment quality: A review of the literature. Build. Environ..

[2] Klepeis N.E., Nelson W.C., Ott W.R., Robinson J.P., Tsang A.M., Switzer P., Behar J.V., Hern S.C., Engelmann W.H. (2001). The National Human Activity Pattern Survey (NHAPS): A resource for assessing exposure to environmental pollutants. J. Expo. Anal. Environ. Epidemiol..

[3] Šujanová P., Rychtáriková M., Mayor T.S., Hyder A. (2019). A healthy, energy-efficient and comfortable indoor environment, a review. Energies.

[4] Geng Y., Ji W., Wang Z., Lin B., Zhu Y. (2019). A review of operating performance in green buildings: Energy use, indoor environmental quality and occupant satisfaction. Energy Build..

[5] (2018). DIRECTIVE (EU) 2018/844 OF THE EUROPEAN PARLIAMENT AND OF THE COUNCIL of 30 May 2018 amending Directive 2010/31/EU on the Energy Performance of Buildings and Directive 2012/27/EU on Energy Efficiency (Text. with EEA relevance).

[6] Van Hoof J., Mazej M., Hensen J.L.M. (2010). Thermal comfort: Research and Practice. Front. Biosci..

[7] Fabbri K. (2015). A Brief History of Thermal Comfort: From Effective Temperature to Adaptive Thermal Comfort. Indoor Thermal Comfort Perception.

[8] d’Ambrosio Alfano F.R., Palella B.I., Riccio G., Toftum J. (2018). Fifty years of Fanger’s equation: Is there anything to discover yet?. Int. J. Ind. Ergon..

[9] IEA EBC-Annex 79-Occupant Behaviour-Centric Building Design and Operation. http://annex79.iea-ebc.org/.

[10] Park J.Y., Nagy Z. (2018). Comprehensive analysis of the relationship between thermal comfort and building control research—A data-driven literature review. Renew. Sustain. Energy Rev..

[11] Mehmood M.U., Chun D., Zeeshan Z., Han H., Jeon G., Chen K. (2019). A review of the applications of artificial intelligence and big data to buildings for energy-efficiency and a comfortable indoor living environment. Energy Build..

[12] Kim J., Schiavon S., Brager G. (2018). Personal comfort models—A new paradigm in thermal comfort for occupant-centric environmental control. Build. Environ..

[13] Zhang W., Hu W., Wen Y. (2019). Thermal Comfort Modeling for Smart Buildings: A Fine-Grained Deep Learning Approach. IEEE Internet Things J..

[14] Kim J., Zhou Y., Schiavon S., Raftery P., Brager G. (2018). Personal comfort models: Predicting individuals’ thermal preference using occupant heating and cooling behavior and machine learning. Build. Environ..

[15] Li D., Menassa C.C., Kamat V.R. (2017). Personalized human comfort in indoor building environments under diverse conditioning modes. Build. Environ..

[16] Salamone F., Belussi L., Currò C., Danza L., Ghellere M., Guazzi G., Lenzi B., Megale V., Meroni I. (2018). Integrated Method for Personal Thermal Comfort Assessment and Optimization through Users’ Feedback, IoT and Machine Learning: A Case Study. Sensors.

[17] Salamone F., Belussi L., Currò C., Danza L., Ghellere M., Guazzi G., Lenzi B., Megale V., Meroni I. (2018). Application of IoT and Machine Learning techniques for the assessment of thermal comfort perception. Energy Procedia.

[18] Wu Z., Li N., Peng J., Cui H., Liu P., Li H., Li X. (2018). Using an ensemble machine learning methodology-Bagging to predict occupants’ thermal comfort in buildings. Energy Build..

[19] Jiang L., Yao R. (2016). Modelling personal thermal sensations using C-Support Vector Classification (C-SVC) algorithm. Build. Environ..

[20] Peng B., Hsieh S.-J. Data-Driven Thermal Comfort Prediction With Support Vector Machine. Proceedings of the Volume 3: Manufacturing Equipment and Systems.

[21] Dai C., Zhang H., Arens E., Lian Z. (2017). Machine learning approaches to predict thermal demands using skin temperatures: Steady-state conditions. Build. Environ..

[22] Youssef A., Youssef Ali Amer A., Caballero N., Aerts J.-M. (2019). Towards Online Personalized-Monitoring of Human Thermal Sensation Using Machine Learning Approach. Appl. Sci..

[23] Häfner P., Seeßle J., Dücker J., Zienthek M., Szeliga F. Interactive Visualization of Energy Efficiency Concepts Using Virtual Reality. Proceedings of the EuroVR 2014.

[24] Wang Z., He R., Chen K. (2020). Thermal comfort and virtual reality headsets. Appl. Ergon..

[25] Kuliga S.F., Thrash T., Dalton R.C., Hölscher C. (2015). Virtual reality as an empirical research tool—Exploring user experience in a real building and a corresponding virtual model. Comput. Environ. Urban. Syst..

[26] Yeom D., Choi J.-H., Kang S.-H. (2019). Investigation of the physiological differences in the immersive virtual reality environment and real indoor environment: Focused on skin temperature and thermal sensation. Build. Environ..

[27] Yeom D., Choi J.-H., Zhu Y. (2019). Investigation of physiological differences between immersive virtual environment and indoor environment in a building. Indoor Built Environ..

[28] Chinazzo G., Chamilothori K., Wienold J., Andersen M. The effect of short exposure to coloured light on thermal perception: A study using Virtual Reality. Proceedings of the Lux Europa 2017.

[29] Huang S., Scurati G.W., Graziosi S. (2019). Effects of Coloured Ambient Light on Perceived Temperature for Energy Efficiency: A Preliminary Study in Virtual Reality. http://lensconference3.org/index.php/program/presentations/item/59-effects-of-coloured-ambient-light-on-perceived-temperature-for-energy-efficiency-a-preliminary-study-in-virtual-reality.

[30] Fanger P.O., Breum N.O., Jerking E. (1977). Can Colour and Noise Influence Man’s Thermal Comfort?. Ergonomics.

[31] Bennett C.A., Rey P. (1972). What’s So Hot about Red?. Hum. Factors J. Hum. Factors Ergon. Soc..

[32] Huebner G.M., Shipworth D.T., Gauthier S., Witzel C., Raynham P., Chan W. (2016). Saving energy with light? Experimental studies assessing the impact of colour temperature on thermal comfort. Energy Res. Soc. Sci..

[33] Ziat M., Balcer C.A., Shirtz A., Rolison T. (2016). A Century Later, the Hue-Heat Hypothesis: Does Color Truly Affect Temperature Perception?.

[34] Wang H., Liu G., Hu S., Liu C. (2018). Experimental investigation about thermal effect of colour on thermal sensation and comfort. Energy Build..

[35] Toftum J., Thorseth A., Markvart J., Logadóttir Á. (2018). Occupant response to different correlated colour temperatures of white LED lighting. Build. Environ..

[36] Golasi I., Salata F., de Lieto Vollaro E., Peña-García A. (2019). Influence of lighting colour temperature on indoor thermal perception: A strategy to save energy from the HVAC installations. Energy Build..

[37] Real-Time Physiological Signals | E4 EDA/GSR Sensor. https://www.empatica.com/en-eu/research/e4/.

[38] Taylor S., Jaques N., Chen W., Fedor S., Sano A., Picard R. Automatic identification of artifacts in electrodermal activity data. Proceedings of the 37th Annual International Conference of the IEEE Engineering in Medicine and Biology Society (EMBC).

[39] EDA Explorer. https://eda-explorer.media.mit.edu/.

[40] Kingma B.R.M., Schweiker M., Wagner A., van Marken Lichtenbelt W.D. (2017). Exploring internal body heat balance to understand thermal sensation. Build. Res. Inf..

[41] Marcel Schweiker Comf: Functions for Thermal Comfort Research. https://cran.r-project.org/package=comf.

[42] Schweiker M. (2016). comf: An R Package for Thermal Comfort Studies. R J..

[43] Decree of the President of the Republic of Italy DPR 412/93. http://www.normattiva.it/urires/N2Ls?urn:nir:stato:legge:1993-08-26;412.

[44] ANSI/ASHRAE Standard 55—Thermal Environmental Conditions for Human Occupancy. https://www.ashrae.org/technical-resources/bookstore/standard-55-thermal-environmental-conditions-for-human-occupancy.

[45] OpenFOAM ®-Official Home of The Open Source Computational Fluid Dynamics (CFD) Toolbox. https://www.openfoam.com/.

[46] Roudsari M.S., Pak M. Ladybug: A parametric environmental plugin for grasshopper to help designers create an environmentally-conscious design. Proceedings of the 13 th Conference of International Building Performance Simulation Association.

[47] Cosma A.C., Simha R. (2018). Thermal comfort modeling in transient conditions using real-time local body temperature extraction with a thermographic camera. Build. Environ..

[48] International Organization for Standardization (2005). CEN EN ISO 7730 Ergonomics of the Thermal Environment-Analytical Determination and Interpretation of Thermal Comfort Using Calculation of the PMV and PPD Indices and Local Thermal Comfort Criteria (ISO 7730:2005).

[49] Wu T., Cui W., Cao B., Zhu Y., Ouyang Q. (2016). Measurements of the additional thermal insulation of aircraft seat with clothing ensembles of different seasons. Build. Environ..

[50] Havenith G., Holmér I., Parsons K. (2002). Personal factors in thermal comfort assessment: Clothing properties and metabolic heat production. Energy Build..

[51] Malchaire J., d’Ambrosio Alfano F.R., Palella B.I. (2017). Evaluation of the metabolic rate based on the recording of the heart rate. Ind. Health.

[52] Luo M., Wang Z., Ke K., Cao B., Zhai Y., Zhou X. (2018). Human metabolic rate and thermal comfort in buildings: The problem and challenge. Build. Environ..

[53] Hasan M.H., Alsaleem F., Rafaie M. (2016). Sensitivity study for the PMV thermal comfort model and the use of wearable devices biometric data for metabolic rate estimation. Build. Environ..

[54] Van Craenendonck S., Lauriks L., Vuye C., Kampen J. (2018). A review of human thermal comfort experiments in controlled and semi-controlled environments. Renew. Sustain. Energy Rev..

[55] Mifflin M.D., St Jeor S.T., Hill L.A., Scott B.J., Daugherty S.A., Koh Y.O. (1990). A new predictive equation for resting energy expenditure in healthy individuals. Am. J. Clin. Nutr..

[56] VIVE Hardware. https://www.vive.com/eu/product/.

[57] Unreal Engine 4. https://www.unrealengine.com/en-US/.

[58] Wear Your E4 Wristband—Empatica Support. https://support.empatica.com/hc/en-us/articles/206374015-Wear-your-E4-wristband.

[59] Picard R.W., Fedor S., Ayzenberg Y. (2016). Multiple Arousal Theory and Daily-Life Electrodermal Activity Asymmetry. Emot. Rev..

[60] Bjørhei A., Pedersen F.T., Muhammad S., Tronstad C., Kalvøy H., Wojniusz S., Pabst O., Sütterlin S. (2019). An Investigation on Bilateral Asymmetry in Electrodermal Activity. Front. Behav. Neurosci..

[61] The Correlation Coefficient: Definition. http://www.dmstat1.com/res/TheCorrelationCoefficientDefined.html.

[62] Linear and Quadratic Discriminant Analysis Sklearn’s Documentation. https://scikit-learn.org/stable/modules/lda_qda.html.

[63] LogisticRegression Sklearn’s Documentation. https://scikit-learn.org/stable/modules/generated/sklearn.linear_model.LogisticRegression.html.

[64] Decision Trees-Scikit-Learn 0.21.2 Documentation. https://scikit-learn.org/stable/modules/tree.html.

[65] ExtraTreeClassifier Sklearn’s Documentation. https://scikit-learn.org/stable/modules/generated/sklearn.tree.ExtraTreeClassifier.html.

[66] LinearSVC Sklearn’s Documentation. https://scikit-learn.org/stable/modules/generated/sklearn.svm.LinearSVC.html.

[67] RandomForestClassifier Sklearn’s Documentation. https://scikit-learn.org/stable/modules/generated/sklearn.ensemble.RandomForestClassifier.html.

[68] Accuracy Metric Sklearn’s Documentation. https://scikit-learn.org/stable/modules/generated/sklearn.metrics.accuracy_score.html.

[69] KFold Sklearn’s Documentation. https://scikit-learn.org/stable/modules/generated/sklearn.model_selection.KFold.html.

[70] Recursive Feature Elimination Sklearn’s Documentation. https://scikit-learn.org/stable/modules/generated/sklearn.feature_selection.RFE.html#sklearn.feature_selection.RFE.

[71] Fernández-Delgado M., Cernadas E., Barro S., Amorim D. (2014). Do we need hundreds of classifiers to solve real world classification problems?. J. Mach. Learn. Res..

[72] Geurts P., Ernst D., Wehenkel L. (2006). Extremely randomized trees. Mach. Learn..

[73] Feature selection Sklearn’s Documentation. https://scikit-learn.org/stable/modules/feature_selection.html.

[74] Sklearn.Metrics.Precision_Recall_Fscore_Support-Scikit-Learn 0.21.3 Documentation. https://scikit-learn.org/stable/modules/generated/sklearn.metrics.precision_recall_fscore_support.html.

[75] Chinazzo G., Wienold J., Andersen M. (2019). Daylight affects human thermal perception. Sci. Rep..

[76] Future Home for Future Communities-FHfFC. http://www.fhffc.it/.

